# Features of infllammatory reactions and clinical picture in elderly and young patients with schizophrenia

**DOI:** 10.1192/j.eurpsy.2021.1262

**Published:** 2021-08-13

**Authors:** L. Androsova, A. Barkhatova, V. Sheshenin, S. Zozulya, I. Otman, V. Pochueva, T. Klyushnik

**Affiliations:** 1 Laboratory Of Neuroimmunology, Mental Health Research Centre, Moscow, Russian Federation; 2 Department For The Study Of Endogenous Mental Disorders And Affective States, Mental Health Research Centre, Moscow, Russian Federation; 3 Department Of Gerontological Psychiatry, Mental Health Research Centre, Moscow, Russian Federation

**Keywords:** schizophrenia at young and old age, inflammatory markers

## Abstract

**Introduction:**

It is known that the intensity of inflammation weakens with age, and therefore it is of interest to study the clinical features of schizophreniс process in relation to the level of inflammatory markers.

**Objectives:**

To determine the level of inflammatory markers (the activity of leukocyte elastase (LE) and α1-proteinase inhibitor (α1-PI), autoantibodies (aAB) to neurotrophin S100b and myelin basic protein) in plasma in different years old groups of patients with schizophrenia.

**Methods:**

Two groups of patients with schizophrenia were examined: the 1st group - 19 women aged 60 to 78 years; the 2nd group - 24 women aged 19 to 42 years.

**Results:**

An increase in activity both of LE and α1-PI was found in young patients. This characterizes a balanced inflammatory response. Elderly patients showed a similar increase in the activity of α1-PI, however, LE activity did not exceed the control values. Insufficient LE activity probably characterizes a decrease in the functional activity of neutrophils. The negative correlation was revealed between the activity of LE and TotPsy (PANSS) in the group of elderly patients (r=-0.62, p<05) and positive correlation between aAB to S100b and TotNeg in both groups (r=0.56 and r=0.49, p<05 respectively). There is relationship between age, the activity of psychopathological symptoms and the rate of development of schizophrenia: the rapid course and variety of disorders at a young age, against the poverty of symptoms and a slow rate in the elderly.

**Conclusions:**

There is relationship between the features of inflammatory reactions and clinical picture in elderly and young patients with schizophrenia.

